# Management of a Suspected Renal Cyst Infection With Intracystic Hemorrhage in a Patient With Autosomal Dominant Polycystic Kidney Disease

**DOI:** 10.7759/cureus.39319

**Published:** 2023-05-21

**Authors:** Whitnee Otto, J. Seth Psomiadis, Brandon Kirshner

**Affiliations:** 1 Internal Medicine, Piedmont Macon Medical Center, Macon, USA

**Keywords:** complicated urinary tract infection, hemorrhagic cyst, cyst infection, autosomal-dominant polycystic kidney disease, genetic renal diseases, renal cyst

## Abstract

Renal cyst infections are a serious complication in patients with autosomal dominant polycystic kidney disease (ADPKD). Cyst infections are challenging to treat and have a high incidence of complications such as sepsis and death. No guideline or evidence-based strategy for diagnosis or treatment of cyst infection currently exists. This lack of standardized guidance leads to individualized medical decision-making for each individual case, despite the high risk of morbidity and mortality associated with the infection.

This case describes a 33-year-old female with a past medical history of ADPKD that presented with hematuria, increased urinary frequency, and left flank pain. On computed tomography (CT) imaging, she was found to have a large intracystic hemorrhage with an associated hematoma formation. Laboratory evaluation was remarkable for leukocytosis with left shift but normal renal function. Urinalysis displayed hematuria and the presence of protein, but the culture resulted in no growth. In the presence of clinical signs of infection, she was suspected to have an infected renal cyst that did not have glomerular communication, given the bland urinalysis and negative urine culture. Her hemoglobin stabilized, and she did not require embolization or percutaneous drainage of the cyst. Intravenous levofloxacin was initiated, and the patient clinically improved with the normalization of leukocytosis. Blood cultures remained negative, and she was discharged to home with a course of oral levofloxacin with a resolution of symptoms.

## Introduction

Autosomal dominant polycystic kidney disease (ADPKD) is a condition that presents as numerous renal cysts on bilateral kidneys, which can impair functionality over time. ADPKD is a relatively common condition with a prevalence of approximately one in 2459 [1]. Patients with complications related to ADPKD often present in the acute care setting with hypertension, flank pain, and hematuria and experience a higher incidence of urinary tract infection requiring hospitalization. Hematuria is often the leading complaint at the time of presentation, and it is accompanied by a precipitating event such as a urinary tract infection approximately 62% of the time [2]. Such patients have a higher incidence of cystitis with progression to pyelonephritis, renal cyst infections, and perinephric abscess [3]. Additionally, renal cyst infections are often difficult to diagnose and treat, leading to sepsis and increased rates of mortality. There are no current evidence-based guidelines for the management of renal cyst infection, which results in a lack of standardized care.

Female gender, increased age, and recent instrumentation of the urinary system, in addition to the presence of ADPKD, display an increased incidence of renal cyst infection. However, the diagnosis of this infection is complex due to various non-specific clinical signs and imaging findings. Definitive diagnosis requires aspiration of the infected cyst for analysis and culture. Sallée et al. proposed some criteria for presumed cyst infection in the presence of four conditions: flank tenderness, increased C-reactive protein levels greater than 5 mg/dL, temperature greater than 38℃ (100.4℉), and absence of findings consistent with recent intracystic bleeding with density above 25 Hounsfield units (HU) [4]. Although these criteria allow for guidance, they do not take into account the superimposed cyst infection complicating an intracystic hemorrhage or localize the region of such infection.

In this article, we present a unique case of an intracystic hemorrhage with suspected renal cyst infection in a patient with ADPKD. Confirmation of cyst infection by needle aspiration was not available, so individualized management surrounded clinical response to pharmacologic intervention, evaluation of imaging studies, and symptomatic management. This approach allowed for minimally invasive care when the infected cystic contents could not be aspirated for confirmation.

## Case presentation

A 33-year-old female presented with a two-week history of hematuria, increased urinary frequency, and severe left-sided flank pain. The patient described the pain as severe, located in the left flank with radiation to the groin, and worsened with movement. She experienced symptomatic fever and chills, nausea, and multiple episodes of non-bloody, non-bilious emesis. Her past medical history included ADPKD, a history of pelvic inflammatory disease, hypertension, tobacco use, and polysubstance use. On examination, the patient was tachycardic with a heart rate of 110 and was febrile with a temperature of 102℉. Her left costovertebral angle was remarkably tender to light palpation, and the overlying skin was without erythema or ecchymosis. Kidneys were ballotable bilaterally, but the inferior edge of the left kidney could not be palpated. Other general and systemic examinations were unremarkable.

**Figure 1:**
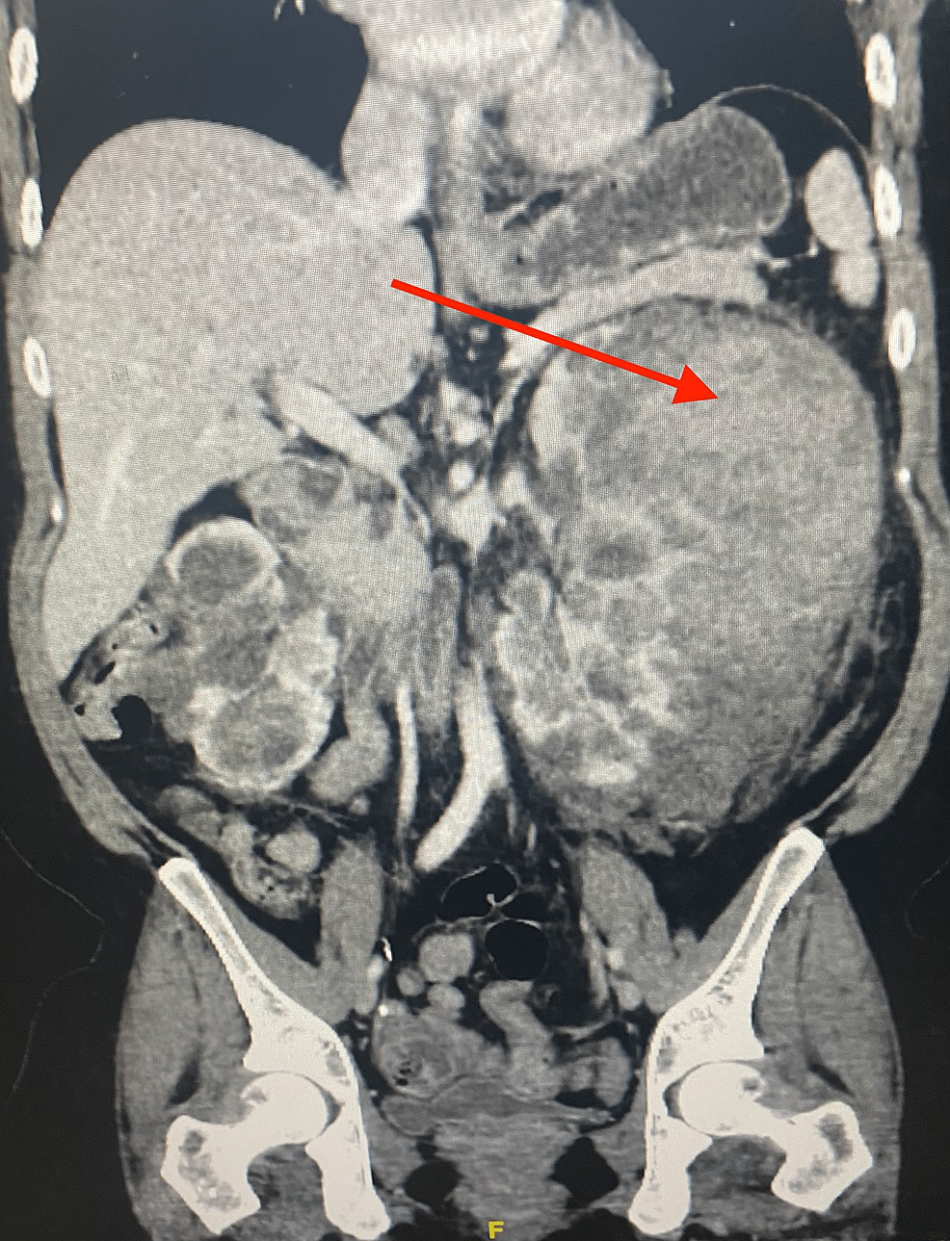
Computerized tomography imaging of bilateral renal cysts in the setting of ADPKD. Bilateral renal enlargement is noted with a contained left-sided intracystic hemorrhage with hematoma formation (red arrow). Left perinephric stranding is seen in addition to the mass effect on surrounding structures. ADPKD: Autosomal dominant polycystic kidney disease.

Her initial laboratory evaluation displayed thrombocytosis of 711 µL and leukocytosis of 25.9 µL with 90.4% neutrophils. Her creatinine was 1.1 mg/dL at presentation, which downtrended with rehydration and remained within normal limits during this admission. A computerized tomography (CT) scan of the abdomen and pelvis with intravenous contrast demonstrated severe bilateral cysts consistent with polycystic kidney disease and left perinephric fat stranding. The left kidney was 11 cm by 14 cm in axial dimension and 18 cm in cranial-caudal direction with localized mass effect and contained a large intracystic hematoma with no radiologic signs of active bleeding. The right kidney was 7 cm by 10 cm in axial dimension and 7 cm in cranial-caudal direction (Figure 1). Urinalysis was positive for protein and hematuria, but urine culture returned negative. One of two initial blood cultures returned positive for a skin flora contaminant (*Staphylococcus epidermidis*), but repeat cultures returned negative and finalized with no growth. Multiple simple hepatic and ovarian cysts were also noted on CT imaging. She was treated empirically with aztreonam due to documented penicillin allergy, which was exchanged for levofloxacin upon admission.

The patient’s hemoglobin decreased from a baseline of 12.3 g/dL to a low of 6.8 g/dL during this admission, requiring transfusion. Repeat CT imaging was completed at this time, which noted slight enlargement of hematoma and capsular distention but no extravasation of contrast. Hemoglobin stabilized after a single unit blood transfusion and began to uptrend to baseline. Although imaging did not display extravasation of contrast at the time of the radiologic exam, there was high clinical suspicion of resolved bleeding due to the substantial drop in hemoglobin and enlargement of the hematoma. No acute bleeding was noted on imaging, and hemoglobin began to uptrend, so embolization was not indicated as determined by interventional radiology.

With rehydration, analgesics, and initiation of antibiotics, the patient symptomatically improved with the normalization of her leukocytosis and improvement in her subjective flank pain. She was discharged to home with a four-week course of levofloxacin 750 mg daily and acetaminophen as needed for pain management. On a two-week follow-up, the patient’s renal function remained at baseline, and she experienced a symptomatic improvement in her flank pain.

## Discussion

We report a case of suspected renal cyst infection in a patient with normal renal function in ADPKD. This case is unique, given the presence of a suspected renal cyst infection with co-existent intracystic hemorrhage and two conditions that are difficult to differentiate clinically. Numerous cases of intracystic hemorrhage in the setting of ADPKD have been reported. Sudden flank pain without a history of trauma is the most common symptom, but other presenting symptoms may include palpable mass, hematuria, anemia, fever, nausea, vomiting, and hypovolemic shock. Ultrasound and CT imaging modalities are frequently used to diagnose cystic hemorrhage with a CT scan as the gold standard. Baishya et al. reported that CT imaging had a 100% sensitivity for diagnosis [5]. Ultrasound can be used; however, the findings can be indistinguishable from a renal mass, leading to misdiagnosis. Differentiation between intracystic hemorrhage and renal cyst infection is difficult with CT imaging alone. However, for renal cyst infections, the use of positron emission tomography-computed tomography (PET-CT) is gaining popularity due to the ability to localize and differentiate infected versus uninfected renal cysts [6]. In this case, the presence of enlarging cyst on imaging and the acute decrease in hemoglobin suggest cystic hemorrhage. The bland urinalysis, as well as the leukocytosis, fever, and significant improvement with antibiotics also suggested the presence of a renal cyst infection.

Sallée et al. described that complicated urinary tract infections in patients with ADPKD are typically caused by gram-negative bacteria. Such infections often ascend from the bladder, presenting initially as cystitis and leading to pyelonephritis. In renal cyst infections, the bacteria are also typically gram-negative, but the source is less clear and may be via hematogenous spread. This can lead to infection of cysts that are not in connection with the collecting system, leading to a bland urinalysis and negative culture despite the presence of infection. Urinalysis of patients with pyelonephritis often is remarkable for white cell casts and the presence of a positive urine culture; however, the urine may be unremarkable in renal cyst infections [4]. This disconnect also leads to challenges in the diagnosis and delays in the treatment of cyst infections.

Antibiotic selection in complicated urinary tract infections is often determined based on a variety of patient factors including drug excretory pathway, cost, efficacy, and infecting organism. Specifically in renal cyst infection, selecting an appropriate drug that achieves both adequate cystic and serum concentrations is essential. A study by Grantham et al. described that cysts over 2 cm are not in communication with the glomerulus, so antibiotics are needed to access the cyst by a mechanism other than glomerular filtration to penetrate [7]. Therefore, the mechanism of action, as well as the localization of infection, is important to consider as most antibiotics cannot access non-communicating cysts and abscesses. Oftentimes, differentiation between pyelonephritis and renal cyst infection is clinically difficult without advanced imaging as discussed previously. Therefore, the selection of empiric antibiotics with coverage in both instances is important. In patients who are not critically ill and have a low risk of multidrug-resistant organisms, fluoroquinolones and trimethoprim-sulfamethoxazole are typically used. Both antibiotics are lipid-soluble, so they can penetrate into a cyst without communicating with the collecting system.

Although cyst aspiration is optimal for bacterial culture and speciation, percutaneous drainage is not always optimal due to multiple factors such as the location of the renal cyst and access to interventional specialists. In this case, the patient had significant clinical improvement with intravenous antibiotics, pain management, and rehydration. She was initially placed on aztreonam but was transitioned to levofloxacin for improved cystic penetration. Levofloxacin also allowed for once-daily oral dosing without the need for a course of intravenous home antibiotics. The patient was discharged home with a course of 21 days of oral levofloxacin. Although symptomatic overlap occurs between cystic hemorrhage and renal cyst infection, both should be individually considered in the evaluation and management of such patients.

## Conclusions

When evaluating a patient with flank pain, fever, and bland urinalysis, it is important to consider renal cyst infection even in the coexistence of a hemorrhagic renal cyst. The presence of fever, flank pain, and elevated inflammatory markers suggest the presence of infection; however, these findings can also be present in intracystic bleeding, making the differentiation difficult. The use of advanced imaging techniques, such as PET scans, is helpful; however, their availability is limited. If there is suspicion of cyst infection, antibiotic coverage must take cyst penetrance and glomerular communication into account. Although the management of such infections can be complex in nature, early identification can reduce complications and allow for conservative management without the need for immediate intervention.
